# From feed coal to fly ash: how transient oil co-firing reshapes hazardous-element budgets, sulphur balance and leaching behaviour of power-plant ashes

**DOI:** 10.1007/s10653-026-03561-6

**Published:** 2026-10-30

**Authors:** Luis F. O. Silva

**Affiliations:** Department of Civil and Environmental Engineering, Universidad de la Costa, Calle 58 #55-66, Barranquilla, 080002 Atlántico Colombia

**Keywords:** Pulverised-fuel combustion, Ash petrology, Trace-element mobility, Oil co-firing, Element leaching, Fly ash atmospheric emission

## Abstract

Feed coals, fly ashes and bottom ashes were collected simultaneously from seven pulverised-fuel units of a large Brazilian thermoelectric complex and examined by combined petrographic, mineralogical and geochemical methods in order to trace the links between the inorganic constituents of the fuel and those of the resulting combustion residues. Two of the units were sampled while their boilers were being started up with co-fired fuel and diesel oil, which provided an opportunity to assess how oil co-firing alters the residues. The feed coals proved remarkably uniform in mineral matter and bulk ash chemistry across the seven units, and the crystalline assemblage of the ashes, dominated by quartz, mullite and maghemite within a large amorphous fraction, was consistent with thermal transformation of that mineral matter. The fly ashes generated during oil co-firing were distinguished by markedly higher contents of unburnt carbon and retained sulphur, which reflect less complete combustion and lower furnace temperatures during start-up. These same ashes were enriched in several moderately volatile trace elements, with arsenic and mercury increasing by more than an order of magnitude. Principal component analysis of the trace-element budget resolved the normally fired ashes, the co-fired ashes and the bottom ashes as discrete populations, and the loadings ordered the elements along a volatility and chalcophile affinity gradient. In water-based leaching tests the co-fired fly ashes produced acidic conditions, in contrast to the alkaline leachates of the normally fired fly ashes, a difference governed by the balance between sulphate and the alkaline-earth oxides. Cationic elements were consequently more mobile from the acidic co-fired ashes, whereas elements released as oxy-anions remained more mobile from the alkaline ashes despite comparable or higher total contents. The work shows that a transient operational practice such as oil co-firing during start-up can measurably change the carbon content, sulphur retention, trace-element budget and leaching response of the ash produced. The dataset is reinterpreted here with an environmental focus, considering leaching from open coal stockpiles held for months on site, the small but element-rich fraction of fly ash that escapes capture and handling and reaches the atmosphere despite extensive reuse in cement, and the large bottom-ash deposits that remain incompletely utilised around the station.

## Introduction

Brazil holds one of the largest electricity-generating capacities in the Western Hemisphere, yet its supply rests mainly on hydropower and, to a smaller degree, petroleum, with coal contributing only a modest share. About 1500 MW, close to one tenth of national generation, is produced by a small group of coal-fired stations concentrated in the southern states of Rio Grande do Sul, Santa Catarina and Paraná, which together discharge on the order of three million tonnes of ash each year, most of it as fly ash (Energy Information Administration, 2010; Levandowski & Kalkreuth, 2008). The coals burned in these plants are typically high in ash and sulphur, and the limited efficiency of fine-particle control has long raised concern over the release of respirable material (Silva et al., 2009a; Teixeira et al., 2009).

Although coals and combustion products from Brazil and elsewhere have been characterised mineralogically and geochemically in numerous studies, comparatively few have followed the inorganic constituents from the feed coal through to the resulting residues (Depoi et al., 2008; Pires & Querol, 2004; Querol et al., 1997; Silva et al., 2009b; Vassilev & Vassileva, 1997). The continuing relevance of this fuel-to-residue tracing is underscored by recent work documenting persistent soil and ecosystem contamination in the vicinity of thermal power plants long after combustion, including potentially toxic elements transported and deposited beyond the immediate plant footprint, organic pollutants such as polycyclic aromatic hydrocarbons whose speciation varies with fuel type, and the migration of leachable metals into surface waters and aquatic food chains (Gabov et al., 2024; Kravchenko et al., 2025; Luo et al., 2024; Park et al., 2023; Ribeiro et al., 2025; Vig et al., 2023). A further obstacle is that most Brazilian stations burn blends drawn from several mines, the proportions of which are not routinely recorded, so that the characteristics of the combustion products are difficult to anticipate or to attribute to a given source (Levandowski & Kalkreuth, 2008).

The present study integrates petrographic, mineralogical and geochemical data on the feed coal, fly ash and bottom ash from the individual units of the Jorge Lacerda complex in Santa Catarina, the largest coal-fired station in South America. Fly ash from this region is used mainly in cement and concrete, with smaller quantities employed as a road-base stabiliser and in asphalt, glass composites and ceramics, whereas bottom ash is generally landfilled or deposited at nearby disused mine sites, where uncontrolled dumping may expose it to leaching and the release of pollutants (Depoi et al., 2008; Silva & da Boit, 2010; Triffault-Bouchet et al., 2005). Beyond their environmental liability, these residues are increasingly viewed as secondary geochemical reservoirs, since coal ashes concentrate critical and trace metals, including rare-earth elements, in proportions that can substantially exceed those of the parent coal (Dai et al., 2025; Mkhize et al., 2026; Sun et al., 2026).

At the time of sampling, two of the units were being started up with a mixture of fuel and diesel oil, which allowed the influence of oil co-firing on the residues to be examined. Co-firing of coal with petroleum coke, sewage sludge and various forms of biomass has been studied before, but the brief episodes of oil co-firing that accompany boiler start-up have received little attention even though they may leave a measurable imprint on the ash (Hower et al., 2005; Izquierdo et al., 2007, 2008; Navarrete et al., 2004). The aims here were therefore to relate the mineralogy and chemistry of the feed coals to those of their ashes and to establish how the transient oil co-firing modifies the composition and leaching behaviour of the residues.

## Materials and methods

The mineralogical, geochemical, petrographic and leaching dataset reinterpreted in the present work was generated for the feed coals and ashes of the Jorge Lacerda complex and is analysed here with an environmental emphasis; the original measurements and full analytical protocols are reported by Silva et al. (2010a), and the salient procedures are summarised below. The station comprises seven pulverised-fuel units, commissioned progressively between 1965 and 1997, with rated capacities ranging from 50 to 363 MW. Feed coal, fly ash and bottom ash were collected at the same time from each unit over a five-day campaign following ASTM D 2234-89, with fly ash drawn from the respective electrostatic precipitators. The fly and bottom ashes of two units were obtained while their boilers were being started up with co-fired oil, and these samples are identified throughout as the co-fired materials. Around 15–20 kg of each ash and about 2 kg of coal were taken per unit, quartered and sub-sampled, and the bottom ash fractions were milled before analysis (Querol et al., 1997). During these start-ups the diesel oil was co-fired with the pulverised coal at no fixed oil–coal ratio: the proportion of diesel is progressively reduced as the furnace heats up and is governed by the quality of the coal, its moisture content and related feed characteristics rather than by a set value. According to the plant operators, once the furnace reaches approximately 900–1100 °C the coal sustains combustion unaided and diesel firing is discontinued, so the oil-assisted phase is confined to the early, lower-temperature part of each start-up. The co-fired fly and bottom ashes analysed here are the residues produced during this oil-assisted start-up phase, collected over the same five-day campaign.

Polished particulate pellets of each coal were examined under reflected light, with maceral nomenclature following the International Committee for Coal and Organic Petrology and vitrinite reflectance measured at 546 nm; pulverised vitrinite could not be subdivided, so only total vitrinite is reported (International Committee for Coal & Organic Petrology, 1998, 2001). Fly ash petrography was carried out on Sudan Black-stained pellets using the scheme of Hower et al. (1995). The mineralogy of the coals was assessed on low-temperature oxygen-plasma ash by X-ray powder diffraction, with quantitative phase proportions obtained by Rietveld-based refinement using commercial software, and the same diffraction approach, incorporating a poorly crystalline silica standard to estimate the non-crystalline content, was applied to the powdered ashes (Gluskoter, 1965; Taylor, 1991; Ward & French, 2006).

Major-element compositions were determined by X-ray fluorescence on fused disks prepared from coal ash, fly ash and bottom ash, with loss on ignition measured during preparation (Norrish & Hutton, 1969). The solid samples were digested by a two-stage procedure designed to retain volatile elements, combining a hot nitric-acid extraction with a hydrofluoric-nitric-perchloric attack of the residue, and the resulting solutions were analysed by inductively coupled plasma atomic-emission and mass spectrometry; an international coal reference material and blanks were processed identically to verify accuracy, and mercury was measured directly on the solids by gold-amalgam atomic absorption (Querol et al., 1997). Element mobility was evaluated with the European standard compliance leaching test EN 12457-2, a single batch extraction at a liquid–solid ratio of ten with deionised water, performed in duplicate, the leachates being analysed by the same spectrometric techniques.

The multivariate and bivariate treatment applied here to the reinterpreted dataset used standard chemometric procedures. Trace-element concentrations were base-ten logarithmically transformed to accommodate their approximately log-normal distribution, then autoscaled to unit variance before principal component analysis, so that the ordination reflects covariance structure rather than absolute abundance (Tian & Zhao, 2024). Enrichment factors were computed as the ratio of the mean concentration in the two co-fired ashes to the mean in the five normally fired ashes, and inter-element and element-carbon associations were quantified by Pearson correlation; the acid–base index SO_3_/(CaO + MgO) was calculated on a loss-free oxide basis and regressed against leachate pH (Cornelis et al., 2008; Ward et al., 2009).

## Results and discussion

### Feed coals

#### Petrology

The fuel is a blend of several sources and is, in general, high-volatile C to high-volatile A bituminous in rank (that is, spanning the high-volatile C, B and A subdivisions of the ASTM D388 rank classification), with maximum vitrinite reflectance between about 0.51% and 0.79% and rare lower-rank components, so that individual units may receive slightly different proportions of the blend. The coals carry moderate and roughly equal amounts of vitrinite and of fusinite plus semifusinite, an inertinite-rich character typical of Gondwana coals, together with a distinct component of petroleum or metallurgical coke; the presence of inertinite within some coke particles suggests that coal-derived coke, possibly a coke-breeze by-product, forms part of the fuel and helps to explain the anisotropic coke later found in the fly ash (Taylor et al., 1998). Pyrite is the dominant sulphide, occurring as framboids, cell and cavity infillings, replacements and cubic crystals, while pyrrhotite was detected only as an accessory phase in the coke particles.

#### Mineral matter

**Fig. 1:**
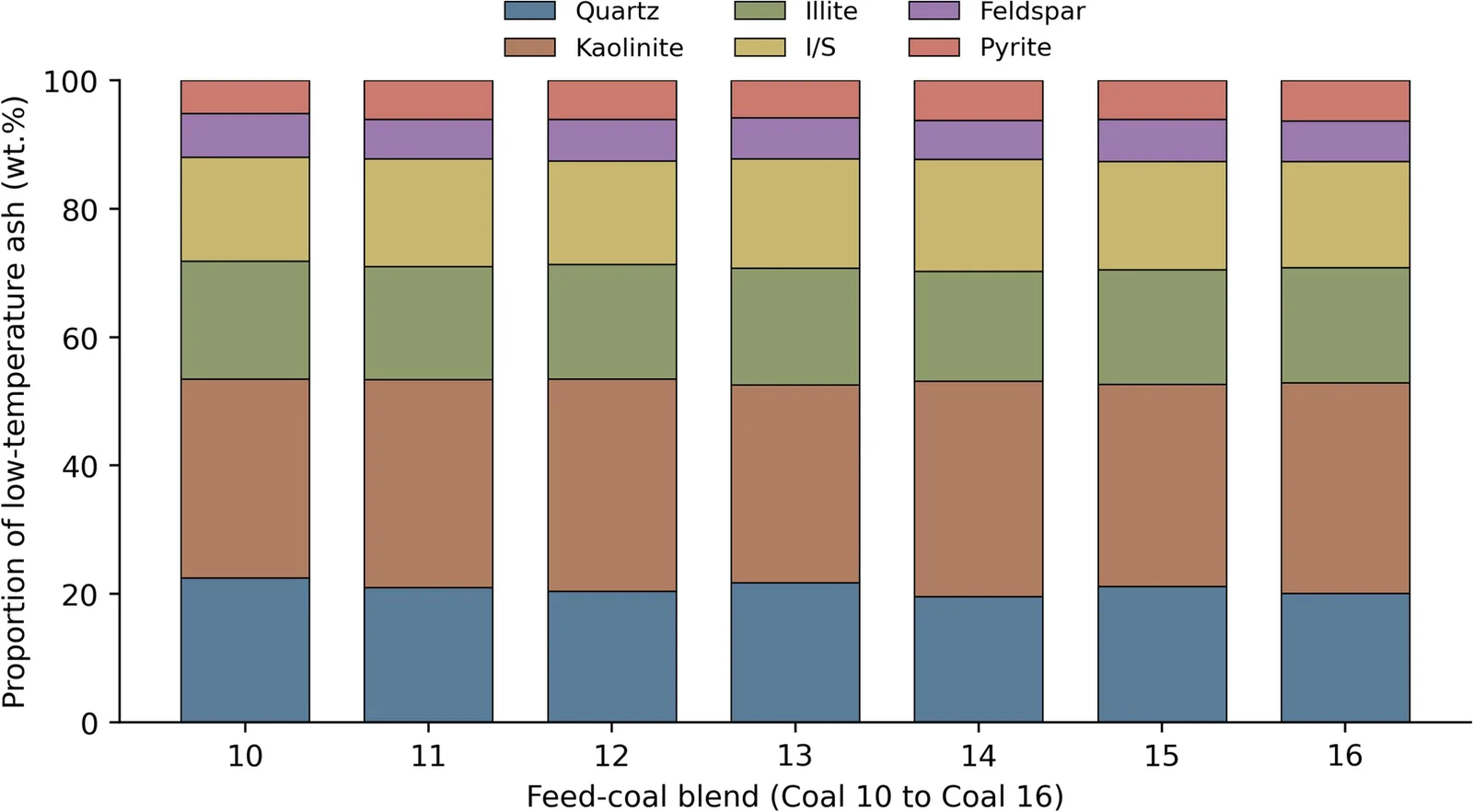
Mineralogical composition of the low-temperature ash of the seven feed-coal blends (Coal 10–Coal 16, the numbering being the operator’s own unit identifiers retained from the source dataset of Silva et al. (2010a) so that the labelling remains consistent across the wider study rather than renumbered 1–7 here), expressed as weight % of the low-temperature ash. Only phases present at concentrations of at least 1 wt% are shown; the minor phases calcite, dolomite, anatase and rutile, together with the bassanite artefact of low-temperature ashing, are omitted

The mineral matter of the coals is dominated by quartz, kaolinite, illite and interstratified illite–smectite, with smaller amounts of albite-like feldspar and pyrite and only traces of calcite, dolomite, anatase and rutile; the proportions of these phases, expressed as weight % of the low-temperature ash, are summarised in Fig. 1, where the trace phases below 1% are omitted. A small amount of bassanite in the residues is regarded as an artefact of low-temperature ashing, formed by reaction between organically associated calcium and sulphur released during oxidation of the macerals (Ward, 2002; Ward et al., 2001). The yield of low-temperature ash lay between about 45 and 49%, and both the yield and the phase proportions were closely similar across the seven units, indicating a consistent inorganic input to the different boilers.

The major-element chemistry inferred from the diffraction data agrees closely with the composition of the high-temperature ash prepared from the coals. Silica and alumina derived from the mineral percentages are slightly higher, and iron, calcium and magnesium slightly lower, than in the directly measured ash, a discrepancy that probably reflects assumptions made about the compositions of the clay components and the presence of amorphous iron oxides not detected by diffraction (Ward et al., 2001). The directly measured ashes themselves were rich in silica, at about 57–62%, and alumina, at about 25–27%, with a few per cent of iron oxide and only minor alkaline-earth and alkali oxides, and were again very similar from one unit to another.

#### Trace elements

Trace-element contents of the feed coals are for the most part comparable with the ranges reported for coals worldwide, although chromium, gallium, thorium, uranium, vanadium and zirconium are somewhat elevated (Dai et al., 2025; Finkelman, 1994; Ren et al., 1999; Swaine, 1990). These higher levels are most plausibly linked to the high proportion of clay minerals in the coals and in associated cleaning rejects, while the petroleum coke in the blend contributes a minor additional input of vanadium and nickel (Hower et al., 2005; Silva et al., 2010b). With the exceptions of boron, zinc and germanium, the seven coals showed no marked differences from one another, reinforcing the picture of a uniform inorganic feed across the units.

### Fly ash

#### Mineralogy

Quartz and mullite are the principal crystalline phases of the fly ash, accompanied by maghemite and, in a few samples, traces of anhydrite, rutile and hematite, all set within a large amorphous fraction that accounts for roughly 60–70% of the material. Comparison with the feed-coal mineralogy indicates that most of the mullite and glass arose from thermal reaction of the clay minerals, that maghemite is the residue of pyrite breakdown, and that the quartz represents relict grains that survived combustion, its lower abundance in the ash than in the coal showing that some quartz was taken up into the melt (Matjie et al., 2008; Ward & French, 2006). The two co-fired fly ashes are distinctive in containing measurable anhydrite and a high proportion of unburnt carbon, the anhydrite being consistent with condensation of calcium sulphate on particle surfaces during the cooler start-up combustion (Brownfield, 2002).

#### Petrology

Optical examination shows that the fly ash is overwhelmingly glassy, glass accounting for roughly 79–94% by volume, with subordinate iron-oxide spinels such as magnetite, hematite and maghemite, isotropic and anisotropic coke, inertinite associated with the anisotropic coke, and partially fused rock fragments whose outer rims have vitrified while their clay-rich interiors remain only partly altered. Most of the normally fired ashes are comparatively coarse, whereas the two co-fired ashes contain conspicuously more microscopically visible carbon, one of them showing fine glass together with coarse carbon-glass aggregates, in keeping with the less efficient combustion during oil-assisted start-up (Hower et al., 1995). The unburnt carbon of the co-fired ashes is petrographically heterogeneous, and the two provenances can be told apart to a first approximation on morphological grounds: char derived from the coal macerals retains recognisable botanical or inertinite-related textures and is associated with the anisotropic and isotropic coke already noted in the feed, whereas the oil-derived component appears as smooth, dense, often spherical soot and cenospheric char lacking internal cellular structure. A rigorous partition of the two would require complementary carbon-isotope or programmed-oxidation analysis, which lies beyond the present dataset, so the distinction is here made on optical grounds only.

#### Major and trace elements

On a loss-free basis the major-element compositions of the fly ashes are mutually similar and close to those of the laboratory ashes of the feed coals, as expected given that fly ash makes up more than nine tenths of the solid residue. The two co-fired ashes stand apart, however, in their much higher loss on ignition and carbon contents and in a modest enrichment in sulphur, expressed as sulphur trioxide, which accompanies the small amounts of anhydrite noted above. The fuel and diesel oils used during the oil-assisted start-up carried only 0.02% and 0.05% sulphur respectively, so the oils were not themselves an important source of the condensed sulphur, which is instead attributed to the cooler combustion favouring retention of sulphur oxides on the particle surfaces (Brownfield, 2002; Navarrete et al., 2004).

**Fig. 2:**
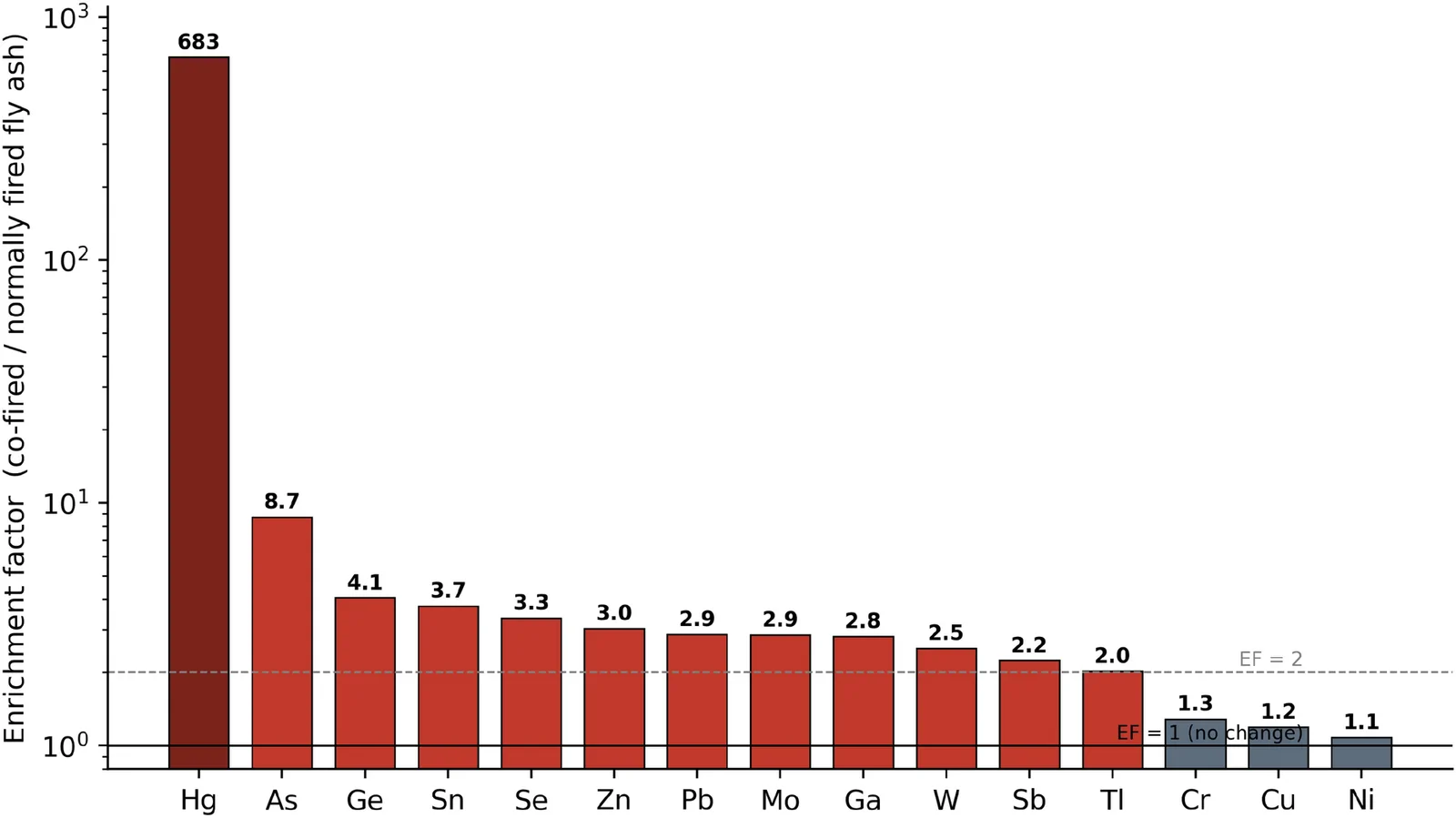
Enrichment factors of the trace elements in the co-fired fly ashes relative to the normally fired fly ashes, expressed as the ratio of the mean concentration in the two co-fired ashes to the mean in the five normally fired ashes, on a logarithmic ordinate. The horizontal solid line marks the absence of enrichment and the dashed line a twofold enrichment. Dark bars denote enrichment exceeding an order of magnitude, intermediate bars twofold to tenfold enrichment, and grey bars the near-inert chromium, copper and nickel

The trace-element contrast between the two ash populations is most economically expressed as an enrichment factor, the ratio of the mean concentration in the two co-fired ashes to the mean in the five normally fired ashes, which is displayed on a logarithmic ordinate in Fig. 2. The factor is dominated by mercury, which rises by roughly 670-fold, followed by arsenic at 8.7, germanium at 4.1, tin at 3.7, selenium at 3.3, zinc at 3.0, lead and molybdenum at 2.9, gallium at 2.8, tungsten at 2.5, antimony at 2.2 and thallium at 2.0, whereas chromium, copper and nickel barely change at factors of 1.1–1.3. The systematic ordering of the enriched elements, all of moderate volatility and predominantly chalcophile or siderophile in affinity, against the near-inert behaviour of the more lithophile and refractory chromium, copper and nickel, demonstrates that the imprint of oil co-firing is a selective volatility-controlled partitioning rather than a uniform compositional shift. The behaviour is best explained by the lower combustion temperatures of start-up enhancing condensation of the volatilised species onto the cooling ash, an effect amplified by the abundant unburnt carbon, which provides reactive sorptive surfaces for the recondensing vapours (Clarke, 1993; Cowan et al., 2024). Nickel, although relatively abundant in the fuel oil, was not appreciably enriched, and vanadium, strongly enriched in the co-fired ashes, occurred only at low concentrations in the oil, so that the oils were not a significant source of the additional trace elements.

**Fig. 3:**
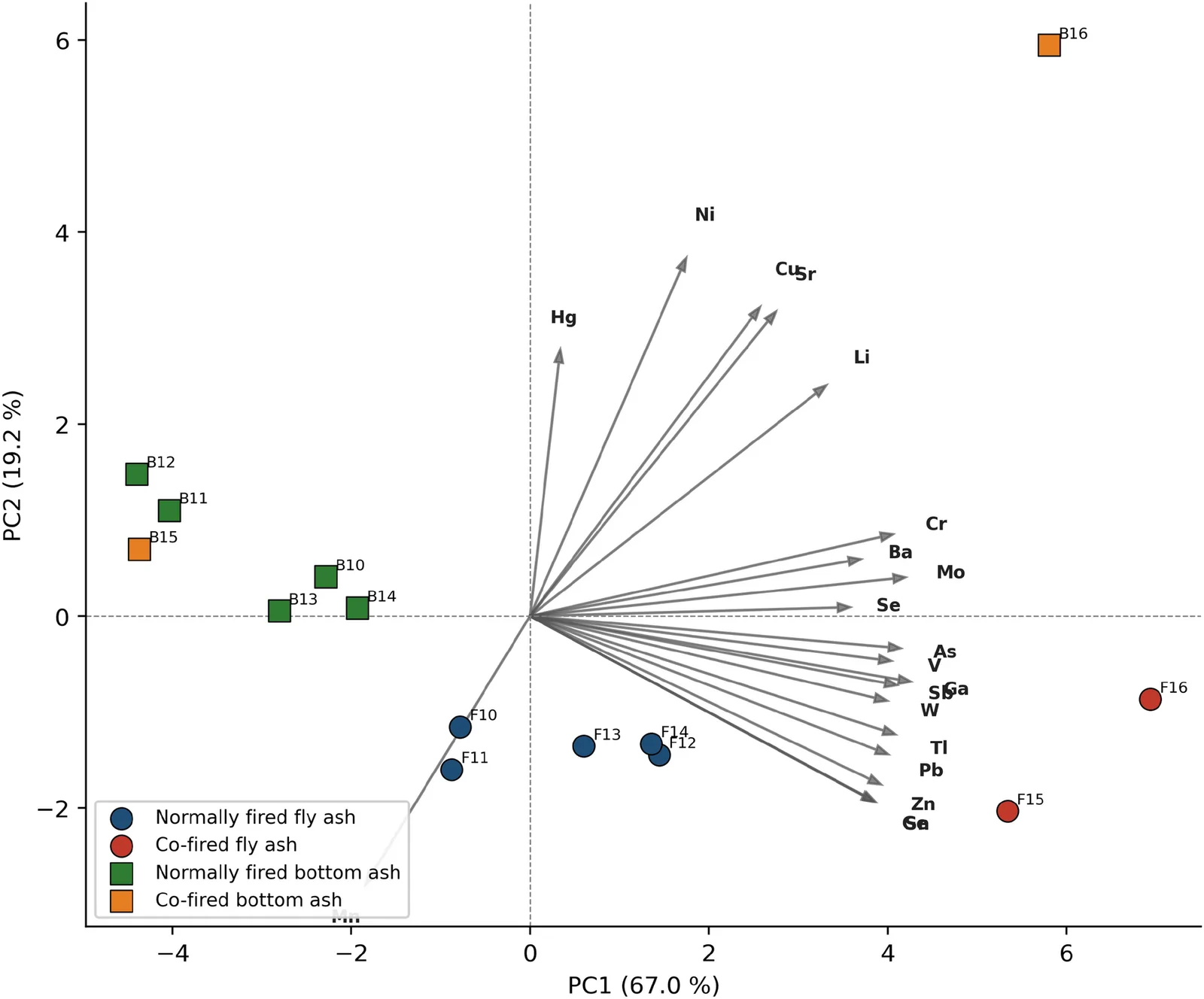
Principal component analysis biplot of the autoscaled, base-ten logarithmically transformed trace-element concentrations of the fly and bottom ashes. Symbols denote the sample groups (circles, fly ash; squares, bottom ash; blue and green, normally fired; red and orange, co-fired) and grey vectors the element loadings. Sample labels are abbreviated (F, fly ash; B, bottom ash). The first component separates the co-fired fly ashes along the volatility and chalcophile affinity gradient, while the second component isolates the iron-segregated bottom ashes

The multivariate structure of the trace-element budget was resolved by principal component analysis of the autoscaled, log-transformed concentrations of the fly and bottom ashes, the ordination of which is shown in Fig. 3. The first two components captured 67.0% and 19.2% of the total variance, and together they segregated the samples into three coherent fields, the normally fired fly ashes, the co-fired fly ashes and the bottom ashes, with the single co-fired bottom ash projecting away from its normally fired counterparts. The first principal component, along which the co-fired fly ashes plot at the positive extreme, carries strong positive loadings for the volatility-affiliated and chalcophile suite, arsenic, mercury, germanium, tin, selenium, zinc, lead, molybdenum, gallium, tungsten, antimony and thallium, so that this axis is interpretable as a condensation and carbon-sorption gradient that quantifies the cumulative imprint of oil co-firing on the residues. The clean separation of the three ash populations along mutually near-orthogonal directions confirms that the co-fired signature is statistically distinct from the inherited lithogenic background that dominates the normally fired and bottom ashes (Liu et al., 2019; Sun et al., 2026).

An interpretation that has rarely been articulated in the ash literature is that these transient start-up episodes constitute a within-station analogue of the temperature-controlled volatility fractionation that is ordinarily inferred only by comparing different boilers or different fuels. Because the seven units share a single uniform feed coal, established here from the near-identical low-temperature ash yields and mineral proportions, the only variable that distinguishes the co-fired from the normally fired residues is the lower furnace temperature and incomplete combustion of the oil-assisted start-up, so the paired sampling isolates the thermal variable in a manner approaching a controlled experiment. Read in this way, the enrichment-factor sequence of Fig. 2 and the first principal component of Fig. 3 are not merely descriptive contrasts but an empirical volatility ranking, in which the position of each element along the condensation gradient encodes the temperature at which its vapour pressure becomes high enough for it to partition out of the melt and recondense onto the cooling, carbon-rich particle surfaces. The internal consistency of this ranking with the classified volatility behaviour reported for full-scale combustion and laboratory heating of coal supports its use as a transferable diagnostic, whereby the appearance of a mercury, arsenic, germanium, selenium and tin signature in an environmental sample could be read back as evidence of low-temperature or oxygen-starved combustion conditions rather than of a change in the fuel itself (Clarke, 1993; Liu et al., 2020; Suárez-Ruiz et al., 2023).

The inter-element and element-carbon association structure underlying the ordination is also evident in the inter-element Pearson correlations. The volatility-affiliated suite forms a tightly intercorrelated cluster, with coefficients commonly exceeding 0.9 among arsenic, germanium, tin, zinc, lead, molybdenum, tungsten, thallium and vanadium, signalling a shared condensation-controlled mode of incorporation. Unburnt carbon correlates positively with this entire suite and, most diagnostically, with mercury at a coefficient near 0.62, whereas nickel stands apart as essentially uncorrelated with the others, consistent with its predominantly lithogenic and oil-borne provenance and its negligible enrichment. The carbon phases of fly ash have long attracted attention for their role in combustion efficiency, ash utilisation and the capture of volatile species, and the very high mercury contents of the co-fired ashes are consistent with mercury interacting with their greater proportion of carbon (Hower et al., 2000, 2010).

**Fig. 4:**
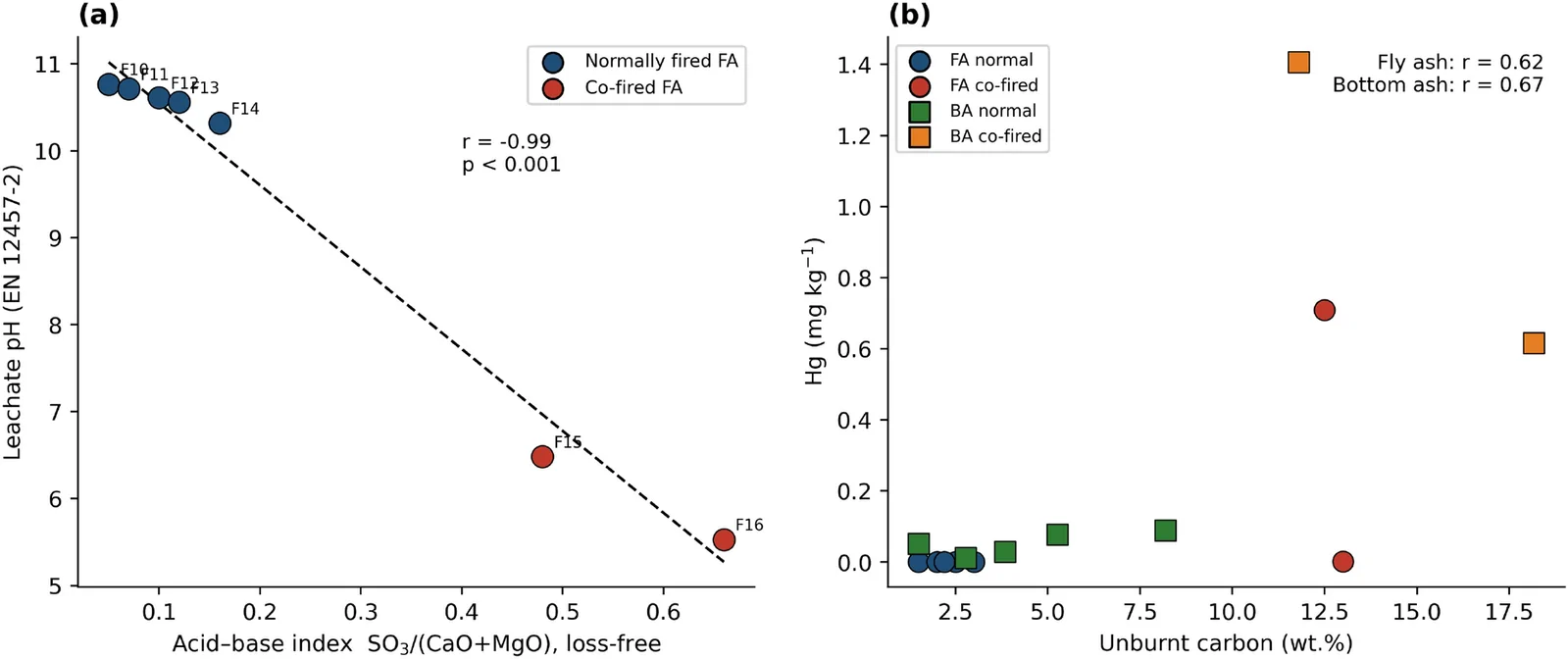
**a** Loss-free acid–base index SO_3_/(CaO + MgO) of the seven fly ashes regressed against the pH of their EN 12457-2 leachates, with the normally fired and co-fired ashes distinguished by colour and the dashed line the least-squares fit (Pearson *r* = − 0.98). **b** Unburnt carbon content against the solid-phase mercury concentration of the fly and bottom ashes (Pearson r = 0.62 and 0.67 respectively), demonstrating the carbon-hosting of mercury in the carbon-rich co-fired residues

Although elements other than mercury are not usually associated with the carbon fraction, arsenic, mercury and selenium have been observed within nanometre-scale metalliferous entities hosted by fullerene-like carbon in a Kentucky fly ash, demonstrating that such associations are possible and that they may be reinforced where oil residues or poorly combusted macerals raise the carbon content of the ash (Gerardo et al., 2023; Hower et al., 2008). Across the present samples this link is borne out quantitatively, the unburnt carbon content correlating positively with mercury, with a Pearson coefficient near 0.64 for the fly ashes and 0.67 for the bottom ashes, as displayed in Fig. 4b, so that the carbon-rich co-fired materials are also those that retain the most mercury.

### Bottom ash

#### Mineralogy

The bottom ashes resemble the fly ashes mineralogically, again being dominated by quartz, mullite and maghemite within a large amorphous fraction. A little anorthite in some samples may point to localised melting and partial recrystallisation at the hotter spots of the combustion system, and the bottom ashes lack the anhydrite and the high unburnt-carbon signatures that distinguish the co-fired fly ashes (Matjie et al., 2008). The near-identical crystalline assemblage of the two residues reflects a shared high-temperature origin, but their separation is essentially physical rather than compositional: the fly ash comprises the fine, low-density fraction (predominantly below about 100 µm, and largely hollow or vesicular glass) that is entrained in the flue gas and carried to the electrostatic precipitators, whereas the coarser, denser and partly agglomerated particles, together with iron-enriched melt droplets, are too heavy to be entrained and fall to the base of the furnace as bottom ash. The slight iron enrichment and the anorthite of the bottom ashes are consistent with this gravitational and thermal segregation, so that particle size, density and degree of melting, rather than mineralogy, govern the split between the two streams.

#### Major and trace elements

On a loss-free basis the bottom ashes carry slightly more iron oxide and slightly less alumina than the corresponding fly ashes, consistent with a tendency for iron to segregate into the coarser bottom material, and their major-element compositions are otherwise comparable; this iron segregation is the geochemical expression of the second principal component in Fig. 3, along which the bottom ashes are displaced. As with the fly ash, the samples collected during oil co-firing contain more unburnt carbon, and loss on ignition tracks the carbon content reasonably well, the residual scatter reflecting the coarse and heterogeneous nature of the bottom ash. Most elements are present at concentrations broadly similar to those of the fly ashes, but the bottom ash formed during co-firing, and one sample in particular, is enriched in a range of volatile-affiliated elements including copper, gallium, germanium, molybdenum, nickel, lead, antimony, selenium, strontium, tin, thallium and zinc, and especially mercury, which again rises by more than an order of magnitude (Rerani et al., 2024). Given the high volatility of mercury, its enrichment in a bottom ash is anomalous and is most readily attributed to association with the elevated carbon content produced by inefficient combustion, a behaviour that warrants further study.

### Leachability of elements

#### pH and major-element mobility

The leachate pH proved to be the master variable controlling element release. The two co-fired fly ashes yielded slightly acidic leachates, with pH between about 5.3 and 6.2, whereas the normally fired fly ashes gave moderately alkaline leachates near pH 9.9–10.4 and the bottom ashes were close to neutral at pH 6.7–7.1. The pH developed when an ash contacts water reflects the balance between its alkaline-earth oxides, which drive alkalinity, and the acid-generating sulphate and phosphate retained on the particles; the acidity of the co-fired ashes follows from their higher sulphur content relative to calcium plus magnesium, reinforced by the formation of sulphuric acid where sulphur dioxide interacts with the ash during humid start-up conditions (Querol et al., 2000; Roy & Griffin, 1984; Ward et al., 2009). When this balance is cast as the loss-free acid–base index SO_3_/(CaO + MgO), it rises from a narrow band of 0.05–0.16 in the five normally fired fly </w:t><w:lastRenderedPageBreak/><w:t>ashes to 0.48 and 0.66 in the two co-fired ashes, and across the seven fly ashes it is very strongly and inversely correlated with leachate pH, the linear regression in Fig. 4a returning a Pearson coefficient of −0.98, which quantifies the dominant control that the sulphate to alkaline-earth stoichiometry exerts on the acid-neutralising capacity of the residue and hence on the pH that develops when the ash meets water (Cornelis et al., 2008). The near-neutral response of the bottom ashes probably reflects stronger incorporation of both calcium and sulphur into the solid structure.

Electrical conductivity told the same story, being lowest for the sparingly soluble bottom ashes, intermediate for the normally fired fly ashes and highest for the co-fired fly ashes, whose abundant condensed sulphate species raised overall solubility. Calcium and sulphur dominated the fly ash leachates and were especially abundant from the co-fired samples, with all other elements released about an order of magnitude lower. Aluminium was more soluble from both the alkaline and the acidic fly ashes than from the near-neutral bottom ashes, in keeping with its amphoteric character, while potassium and magnesium were most mobile from the co-fired ashes and iron was released only at trace levels under acidic conditions and was undetectable above neutral pH (Querol et al., 2000). The magnitude of these releases is comparable with values reported for European and Australian coal fly ashes leached under the same EN 12457-2 protocol, the alkaline normally fired leachates resembling the calcium-sulphate-dominated waters described by Izquierdo and Querol and by Ward et al., while the acidic, higher-conductivity co-fired leachates approach the more aggressive behaviour documented for long-stored acidic ashes (Izquierdo et al., 2008; Querol et al., 2000; Ward et al., 2009).

#### Trace-element mobility

**Fig. 5:**
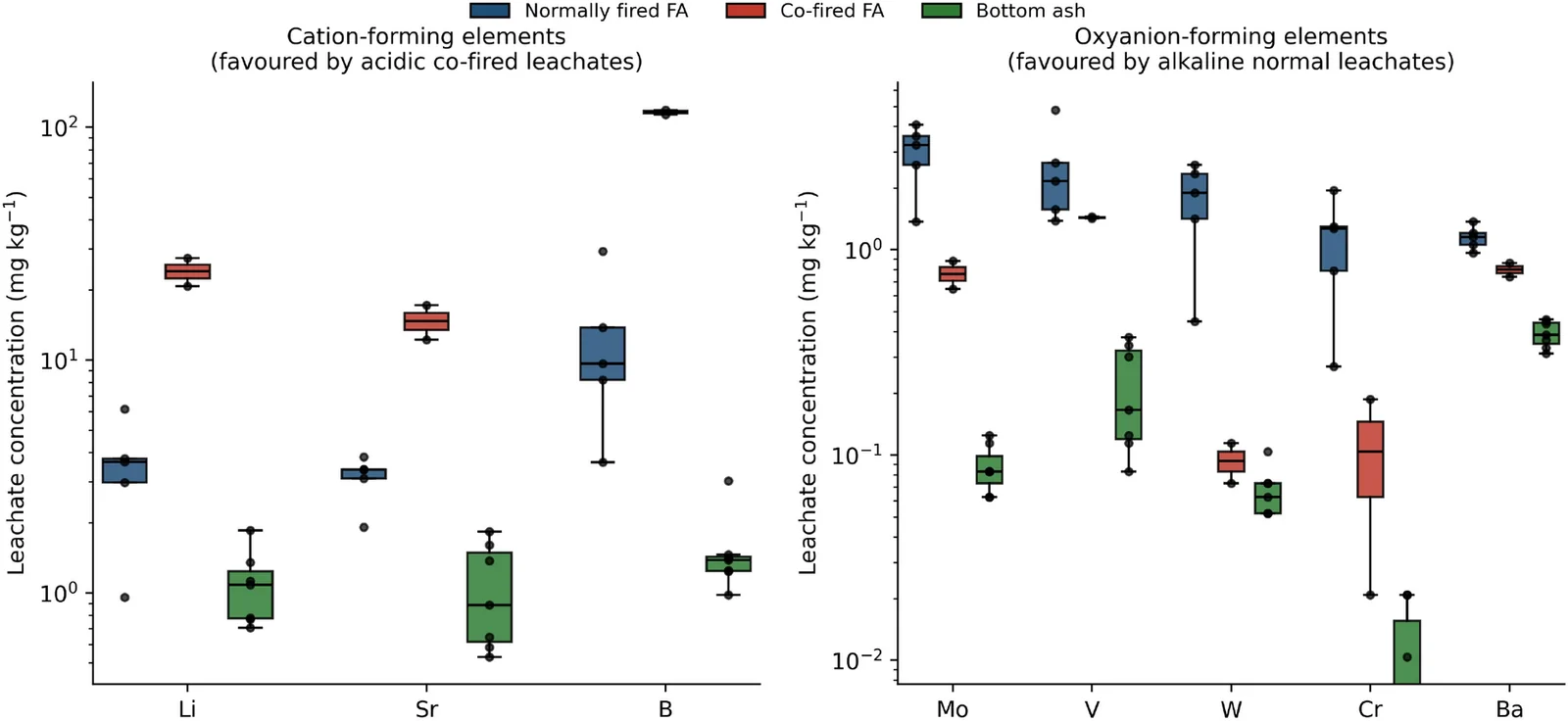
Distribution of selected element concentrations in the EN 12457-2 leachates, grouped as normally fired fly ash, co-fired fly ash and bottom ash, on a logarithmic ordinate, with individual measurements overlaid on the box summaries. The cation-forming elements (left) are preferentially mobilised from the acidic co-fired leachates, whereas the oxyanion-forming elements (right) are preferentially mobilised from the alkaline normally fired leachates, the contrast tracing the pH control imposed by the acid–base index of Fig. 4a

The mobility of the trace elements was governed by leachate pH rather than by their total abundance in the solid, and the contrasting behaviour of the two geochemical families is illustrated in Fig. 5. The cation-forming elements, boron, beryllium, cadmium, cobalt, copper, germanium, lithium, manganese, nickel, rubidium, strontium and zinc, together with several rare-earth elements, were released in higher concentrations from the acidic co-fired fly ashes, behaviour expected of species whose hydrolysis and surface complexation favour dissolution as free or weakly complexed cations under low-pH conditions. By contrast, the oxyanion-forming elements, arsenic, chromium, gallium, molybdenum, vanadium and tungsten, were less mobile from the co-fired ashes than from the normally fired ones, since these elements partition into solution as arsenate, molybdate, vanadate, tungstate, chromate and related oxy-anions whose desorption from amphoteric oxide and hydroxide surfaces is promoted by the high hydroxyl activity of the alkaline normally fired leachates, so that despite comparable or higher total contents they remained relatively immobile in the acidic materials (Cornelis et al., 2008; Jin et al., 2025; Ward et al., 2009). Release from the bottom ashes was generally below detection, reflecting stronger bonding of the elements into the solid phases and a smaller adsorbed fraction on the lower surface area of these coarse materials. Direct comparison of the co-fired and normally fired residues confirms that the contrasts in trace-element mobility, pronounced for the fly ashes, are far more subdued for the bottom ashes. Barium, by contrast, does not form an oxy-anion; as a divalent cation its low mobility from all of the ashes is instead governed by the low solubility of barium sulphate, so that it is grouped here with the pH-independent, solubility-controlled species rather than with the true oxyanion formers.

### Environmental Implications

Beyond the residues themselves, the fuel supply of the station carries an environmental burden that begins before combustion. The coal is held in open stockpiles for prolonged periods, in part because the volumes brought to the plant are tied to regional coal-production arrangements rather than to instantaneous generation demand, so that quantities exceeding the immediate requirement are routinely stored on site. During these months of open-air storage the stockpiles are exposed to rainfall and oxidation, and the high pyrite and clay contents documented above make the coals prone to the acid generation and trace-element release already reported for Brazilian coals and cleaning residues, so that stockpile drainage is a plausible pathway for contaminants to reach soils and surface waters around the plant (Kravchenko et al., 2025; Silva & da Boit, 2010; Silva et al., 2010a).

The fly ash, although directed almost entirely to the cement and concrete industries, is not thereby removed from the atmospheric pathway. The electrostatic precipitators capture close to 98.5% of the fly ash, so that on the order of 1–2% of a very large annual mass escapes directly to the atmosphere, and a further diffuse loss occurs during the open handling and road transport of the ash between the power station and the cement plants (Depoi et al., 2008). The fraction most likely to elude capture is the finest material, which in this study is also the co-fired ash most enriched in arsenic, mercury and other volatile-affiliated elements; because the enrichment factors derived above reach roughly 670 for mercury and nine for arsenic, even a small escaping percentage represents a disproportionate flux of these toxic elements to the surrounding environment, a concern compounded by the demonstrated capacity of fine ash particles to transport and redeposit such elements at distance (Cowan et al., 2024; Hower et al., 2008; Ribeiro et al., 2025).

The bottom ash raises a complementary and longer-term concern, since it is not at present fully utilised and large volumes, amounting to thousands of tonnes, accumulate in stockpiles and disused mine workings around the station. Although the compliance test shows that fresh bottom ash releases most elements only at low levels, prolonged weathering of such deposits, and the near-neutral to mildly acidic conditions that can develop as they age, may progressively mobilise the trace elements they contain, as has been observed for bottom ash reused or deposited in the environment elsewhere (Chen et al., 2024; Triffault-Bouchet et al., 2005; Ward et al., 2009). At the same time, the substantial trace-metal inventory of these residues, including the critical and rare-earth elements concentrated during combustion, marks them as candidate secondary resources whose recovery could offset their disposal liability, so that they are better regarded as a reservoir warranting monitoring and valorisation than as an inert by-product (Fu et al., 2024; Mkhize et al., 2026; Sun et al., 2026). Whether these residues meet the thresholds required for their principal uses is therefore a material question: for reuse in cement and concrete the fly ash is expected to satisfy the requirements of EN 450-1 (loss on ignition and reactive-oxide limits) and ASTM C618, whereas suitability for landfill or road-base placement is judged against the EN 12457-2 waste-acceptance criteria (Council Decision 2003/33/EC), whose inert and non-hazardous limits for sulphate and for the mobile oxyanion formers such as molybdenum, chromium and vanadium are the values most likely to be approached by the co-fired ashes. A formal comparison of the present leachate concentrations against these limit values [AUTHOR TO INSERT: confirm which national/EN limits the journal expects and tabulate the exceedances, if any] would establish directly whether the transient co-fired material remains within the acceptance envelope for these applications.

A consequence that is seldom drawn out when ash is considered simultaneously as a contaminant and as a secondary resource is that the two framings impose opposing demands on the same material, and the present samples make this antagonism unusually explicit. The very attributes that render the co-fired residues environmentally problematic, namely their abundant unburnt carbon and their refractory aluminosilicate glass, are also the principal hosts that lock up the elements of economic interest. The carbon that sorbs and sequesters mercury, arsenic and selenium, demonstrated here by the mercury-carbon correlation, is the same phase whose hydrophobicity and high surface area impede the wetting and dissolution of fine particles during hydrometallurgical extraction, while the rare-earth and critical elements concentrated during combustion are predominantly partitioned into the acid-resistant aluminosilicate glass and into micron to submicron crystalline inclusions encapsulated within it, both of which resist direct leaching and are liberated only when the glass network is disrupted by aggressive alkaline roasting (Berti et al., 2025; Ji et al., 2023; Zheng et al., 2024). The implication is a genuine processing trade-off, because the same high-temperature alkaline activation, soda-ash or hydroxide roasting followed by acid leaching, that is required to break the glass and release the lanthanides would simultaneously volatilise or mobilise the very mercury, arsenic and other chalcophile species that the carbon had immobilised, transferring the environmental burden from a stabilised solid into a process stream or off-gas unless it is captured (Pan et al., 2023; Zhang et al., 2025). Recognising that the contaminant-bearing and resource-bearing fractions are physically coextensive, rather than separable populations, argues for an integrated flowsheet in which volatile-element capture is designed into any future critical-element recovery from these residues, and it identifies the carbon-rich co-fired ashes as the fraction where this coupling is most acute (Darmansyah et al., 2023; Fu et al., 2024).

## Conclusions

Despite some variation in their organic petrology, the coal blends supplied to the seven units shared closely similar mineral matter, dominated by quartz, kaolinite, illite and interstratified illite–smectite with minor feldspar and pyrite, and this uniformity carried through to the bulk chemistry of the laboratory ashes. The fly and bottom ashes were likewise alike from one unit to another, with quartz, mullite and maghemite as the main crystalline phases and a large amorphous fraction. The brief episodes of oil co-firing that accompanied boiler start-up nonetheless left a clear imprint, producing fly ash with high unburnt carbon and retained sulphur that reflect less efficient combustion at lower furnace temperatures.

Those co-fired fly ashes were enriched in a range of moderately volatile trace elements, with arsenic and mercury rising by more than an order of magnitude, most likely through enhanced condensation at the lower temperatures combined with sorption onto the abundant carbon. Principal component analysis resolved the normally fired ashes, the co-fired ashes and the bottom ashes as discrete populations and ordered the trace elements along a volatility and chalcophile affinity gradient, while the mercury-carbon correlation confirmed the carbon-hosting of mercury. Because of their higher sulphur relative to the alkaline-earth oxides, the co-fired fly ashes generated acidic leachates rather than the alkaline leachates of the normally fired ashes, the acid–base index being almost perfectly inversely correlated with leachate pH, so that cationic elements became more mobile while elements released as oxy-anions remained more mobile from the alkaline materials despite similar total contents.

Although such ash is produced only briefly, the results show that oil co-firing during start-up can measurably alter the carbon content, sulphur retention, trace-element budget and leaching response of the residues. Placed in an environmental frame, these findings matter beyond the boiler: the open coal stockpiles held for months on site are a plausible source of leachate, the 1–2% of fly ash that escapes the precipitators together with the diffuse losses during transport carries a disproportionate burden of mercury and arsenic precisely in the co-fired material, and the thousands of tonnes of incompletely used bottom ash stored around the station constitute a long-term reservoir of mobilisable trace elements that is also a candidate secondary source of critical metals. Together these pathways argue for treating the entire fuel-to-residue chain, rather than the ash alone, as the unit of environmental management.

## Data Availability

No datasets were generated or analysed during the current study.

## Declarations

### Use of generative AI and AI-assisted technologies

During the preparation of this work the author used an AI-assisted tool to support language editing and text correction, the organization and formatting of references, the checking of compliance with journal requirements, and the standardization of the figures, including the statistical figures, for a consistent presentation. After using this tool the author reviewed and edited the content as needed and takes full responsibility for the content of the publication.
